# Extracellular vesicles and Duchenne muscular dystrophy pathology: Modulators of disease progression

**DOI:** 10.3389/fphys.2023.1130063

**Published:** 2023-02-14

**Authors:** Laura Yedigaryan, Maurilio Sampaolesi

**Affiliations:** ^1^ Translational Cardiomyology Laboratory, Stem Cell and Developmental Biology, Department of Development and Regeneration, KU Leuven, Leuven, Belgium; ^2^ Histology and Medical Embryology Unit, Department of Anatomy, Histology, Forensic Medicine and Orthopaedics, Sapienza University of Rome, Rome, Italy

**Keywords:** extracellular vesicles (EVs), exosomes, muscle regeneration, miRNAs, muscular dystrophies, cardiomyopathy, satellite cells, induced pluripotent stem cells (iPSCs)

## Abstract

Duchenne muscular dystrophy (DMD) is a devastating disorder and is considered to be one of the worst forms of inherited muscular dystrophies. DMD occurs as a result of mutations in the dystrophin gene, leading to progressive muscle fiber degradation and weakness. Although DMD pathology has been studied for many years, there are aspects of disease pathogenesis and progression that have not been thoroughly explored yet. The underlying issue with this is that the development of further effective therapies becomes stalled. It is becoming more evident that extracellular vesicles (EVs) may contribute to DMD pathology. EVs are vesicles secreted by cells that exert a multitude of effects *via* their lipid, protein, and RNA cargo. EV cargo (especially microRNAs) is also said to be a good biomarker for identifying the status of specific pathological processes that occur in dystrophic muscle, such as fibrosis, degeneration, inflammation, adipogenic degeneration, and dilated cardiomyopathy. On the other hand, EVs are becoming more prominent vehicles for custom-engineered cargos. In this review, we will discuss the possible contribution of EVs to DMD pathology, their potential use as biomarkers, and the therapeutic efficacy of both, EV secretion inhibition and custom-engineered cargo delivery.

## 1 Introduction

Duchenne muscular dystrophy (DMD) is an X-linked degenerative disease of cardiac and skeletal muscles, as a consequence of mutations in the dystrophin gene (Yao et al., 2021) (Verhaart & Aartsma-Rus, 2019). The dystrophin protein is a crucial member of the dystrophin glycoprotein complex (Ribeiro et al., 2019). Dystrophin plays a critical role in the integrity, maintenance, and normal functions of muscle cells. Under normal circumstances, muscle degeneration activates muscle-specific stem cells, namely satellite cells, that differentiate into mature muscle cells, contributing to muscle regeneration (Biera et al., 2018) (Bentzinger et al., 2012). Dystrophin has been described to play a vital role in the asymmetrical division of satellite cells as well as their regenerative capacity (Dumont et al., 2015) (Keefe & Kardon, 2015). Therefore, the absence of dystrophin leads to the replacement of degenerated muscle tissues with fibrotic and fat tissues (Emery, 2002). Copious amounts of different therapeutic approaches for treating DMD have been extensively explored. Some of these include exon-skipping oligonucleotides (Aartsma-Rus et al., 2017), anti-inflammatory and anti-fibrotic agents (Heier et al., 2013) (Levi et al., 2015), and stem cell-based therapies such as the transplantation of satellite cells (Arpke et al., 2013), mesoangioblasts (Sampaolesi et al., 2006), pericytes (Valadares et al., 2014), etc. Although results in pre-clinical models were very encouraging, the clinical trial in five DMD patients based upon four consecutive systemic administrations of human leukocyte antigen (HLA)-matched donor cells (from a sibling) showed safety but no efficacy (Cossu et al., 2015). Thus, many research opportunities arose from the exploration of novel strategies to enhance the efficacy of transplanted cells, including signaling molecules that mediate the crosstalk between donor cells and the muscle regenerative niche.

Currently, the topic of extracellular vesicles (EVs) and their involvement in disease promotion and progression is gaining momentum (Gartz et al., 2020). EVs are nanosized vesicles released by cells (Rome et al., 2019). These vesicles are mediators of intercellular communication and are divided into three different types: apoptotic bodies, microvesicles, and exosomes. Research has shown that EVs derived from diseased cells or bodily fluids from patients or animal models, are most often enriched for pathogenic proteins and nucleic acids (Gartz et al., 2020). As a result, there is growing interest in synthesizing novel therapeutics targeting EVs. It is still debatable whether EVs are simply markers of disease or dynamic participants in the pathological advancement of any given disease (Yates et al., 2022). While EVs have already proven to be potential biomarkers and carriers of DMD-improving molecules, there is still much to be discovered in terms of their involvement in DMD pathophysiology (Meng et al., 2022). It has been discovered that microRNAs (miRNAs) may play the biggest role in EV-mediated disease progression. miRNAs are short non-coding RNAs that bind to messenger RNAs (mRNAs), leading to either translational repression or mRNA degradation (Matsuzaka et al., 2016) (Lee & Vasudevan, 2013). The significance of miRNAs in DMD has been studied extensively and EV-enclosed miRNAs are said to represent potentially effective biomarkers. On the other hand, EVs derived from healthy cells have also contributed to the improvement of DMD symptoms (Aminzadeh et al., 2018).

In this review, we wish to delve into the known aspects of EV involvement in DMD pathophysiology, in both types of striated muscle, and as stated previously, the more well-known aspects of EVs in this disease, namely as potential biomarkers and therapeutic agents. We will also briefly discuss potential therapeutic options with EVs and what the future may hold for nanoparticle therapy.

## 2 Duchenne muscular dystrophy

DMD, as an X-linked disorder, is one of the most severe muscle diseases. Unfortunately, there is still no cure for DMD (Verhaart & Aartsma-Rus, 2019). The incidence of DMD is approximately 1 in 5,136 male births (Crisafulli et al., 2020). Already between the ages of 8 and 10, it is difficult for patients to stand and walk, coaxing the need for assistive equipment such as braces or wheelchairs. Most DMD patients pass away due to cardiorespiratory failure (Meyers & Townsend, 2019) (Silva et al., 2019). However, due to improvements in both respiratory and cardiac care, the life expectancy of DMD patients has increased, with patients reaching their 30s, and even some living into their 40s and 50s (Landfeldt et al., 2020). Sadly, the condition is fatal in 100% of cases (Chemello et al., 2020).

Mutations in the dystrophin gene lead to the occurrence of DMD (Monaco et al., 1986) (Hoffman et al., 1987b) (Hoffman et al., 1987a) (Bonilla et al., 1988) (Haidet et al., 2008) (Okubo et al., 2020) and the severity of DMD strongly depends on the mutation type. ‘In-frame’ mutations generate a partially functional dystrophin protein, leading to a less severe form of DMD, namely Becker muscular dystrophy (Kunkel et al., 1986) (Haidet et al., 2008). On the other hand, “out of frame” mutations completely disrupt the reading frame, leading either to the complete lack of dystrophin or an entirely dysfunctional dystrophin (Aravind et al., 2019). This leads to the severe DMD phenotype. The absence of a functional dystrophin increases the fragility of muscle fibers and promotes ongoing cycles of muscle degradation and regeneration (Ribeiro et al., 2019). Under such circumstances, dysfunctional satellite cells, activation of calcium-dependent proteases and overall disruption of calcium homeostasis, mitochondrial disruption, and inflammatory cytokines greatly contribute to muscle being replaced by fibrotic and fatty tissues (Wakayama et al., 1979) (Klingler et al., 2012).

Current DMD therapeutics focus on dystrophin-targeted therapies and targeting the consequential pathological changes (Guiraud & Davies, 2017). Therapies such as cell therapies, gene therapies, and protein replacement therapies are all dystrophin-targeted therapies. Although much research has gone into these methods, significant challenges persist (Chamberlain & Chamberlain, 2017). Primarily, it is very difficult to target all muscle tissues, given the high abundance and the wide distribution of muscle throughout the whole body (Verhaart & Aartsma-Rus, 2019). On the other hand, such therapies may slow down the progression of the disease, however, the restoration of abnormal muscle tissues is unsubstantiated due to the deteriorating nature of DMD. Targeting downstream pathological processes in DMD also represents a promising but a yet not-fully understood therapy strategy. Much must still be researched, particularly for obtaining in-depth information of the secondary pathological changes since the specific mechanisms of such therapies remain obscure.

## 3 Extracellular vesicles and the prominence of their cargo

EVs are single outer membrane-bound vesicles that can be classified according to their size distribution, biogenesis, and function (Gabisonia et al., 2022). Exosomes represent the population of EVs with the smallest diameter, namely from 50 to 150 nm (Heijnen et al., 1999) (Logozzi, et al., 2021). These vesicles are produced *via* the endolysosomal pathway, formed from the invagination of late endosomes, housed in multivesicular bodies (MVBs) and released by exocytosis.

Briefly, MVBs accumulate intraluminal vesicles (ILVs) and may then either fuse with lysosomes and promote ILV destruction, or fuse with the cell membrane and release ILVs in the extracellular space (Huotari & Helenius, 2011). The biogenesis of MVBs may follow either the endosomal sorting complexes required for transport machinery (ESCRT)-dependent or -independent pathway. The ESCRT-dependent pathway consists of a complex machinery, facilitated by four main complexes and some additional proteins. This pathway leads to the formation of ILVs in a stepwise fashion. On the other hand, ESCRT-independent processes are based on the presence of lipid rafts inside the endosomal membranes. These lipid rafts consist of cholesterol and more importantly, sphingolipids, which are converted to ceramide through the action of sphingomyelinase enzymes (Trajkovic, et al., 2008). The coalescence of ceramide forms microdomains that induce budding and subsequent formation of ILVs.

**FIGURE 1:**
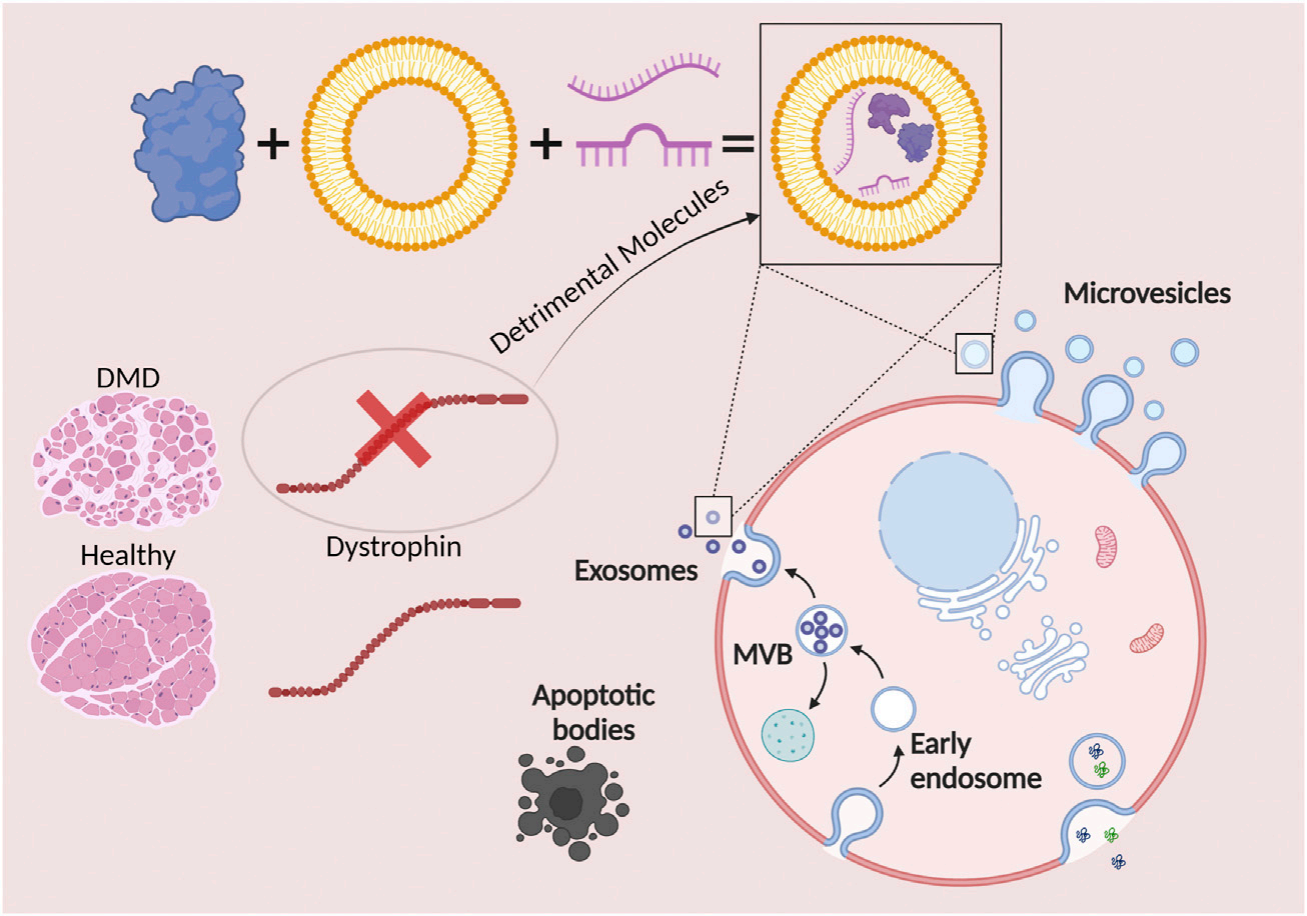
Extracellular vesicles (EVs) are hailed as potential modulators of Duchenne muscular dystrophy progression. EVs are represented by exosomes, microvesicles, and apoptotic bodies. The cargo of exosomes and microvesicles consists of effector molecules such as proteins, lipids, and nucleic acids. The lack of a functional dystrophin leads to the formation and release of exosomes and microvesicles with detrimental molecules, inducing both negative paracrine and endocrine effects.

The next population of EVs are the microvesicles (MVs). These vesicles range from 50 to 1,000 nm in diameter, therefore, being mainly larger than exosomes (Heijnen et al., 1999) (Skog, et al., 2008). MV biogenesis occurs following the direct outward blebbing and pinching of the plasma membrane. Lastly, apoptotic bodies represent the largest (in size) population of EVs, considered to range from 1,000 to 5,000 nm in diameter (Atkin-Smith et al., 2015). As their name suggests, apoptotic bodies are released after programmed cell death, also known as apoptosis (Figure 1).

The cargo of EVs is what makes them incredibly intriguing to study. EVs are known to have a distinctive signature of proteins, lipids, and nucleic acids, analogous to their cell or tissue of origin. EVs are known to be enriched for proteins of the tetraspanin family, namely CD9, CD63, and CD81 (Gabisonia et al., 2022). While still not definitive, these proteins are said to contribute to membrane remodeling to mold the EV structure. EVs are enriched in a multitude of lipids. Although the composition of surface lipids has not been studied extensively, it has been hypothesized that these lipids are crucial for the regulation of EV release, as well as in their final composition (Subra et al., 2007). Lastly, EVs are highly enriched in nucleic acids (Mathivanan et al., 2010), of which mRNAs and miRNAs have been studied comprehensively. In particular, miRNAs are becoming increasingly popular in therapeutic research. miRNAs are non-coding RNAs that either block translation of target mRNAs or activate their degradation (Yedigaryan & Sampaolesi, 2021).

EVs secreted by skeletal and cardiac muscle carry a wide range of myokines, miRNAs, proteins, and mRNAs that are thought to play various roles in muscle homeostasis, development, and myogenesis (Vechetti et al., 2020). Overall, the EV cargo plays a significant role in the musculoskeletal system. In particular, miRNAs and specifically the myomiRs, which are muscle-oriented miRNAs (highly enriched in muscle) (Chen et al., 2006) (Annibalini et al., 2019), display key roles in controlling muscle homeostasis, proliferation, and differentiation of muscle stem cells (Coenen-Stass et al., 2016). As an example, miR-206 is one of the most abundant miRNAs in muscle-derived EVs which aids in muscle injury repair by promoting development and differentiation of tissues (Guescini et al., 2015). These EVs are not only muscle tissue-specific agents but have also been described to affect other tissues (Jalabert et al., 2016) (Rome, 2022). Therefore, much still remains to fully understand the muscle secretome and its extensive range of effects. Although not extensively studied, there has been evidence to show that EVs have an effect on disease pathophysiology. As an example, Gartz et al. showed that treatment with GW4869, a substance to reduce/inhibit exosome release, was protective against cardiac stress in *mdx* mice (a popular model of DMD) (Gartz et al., 2020) (Yates et al., 2022). They attributed this effect to the miRNA cargo. In another study, Matsuzaka et al. showed that the excision of the neutral sphingomyelinase 2/sphingomyelin phosphodiesterase 3 (nSMase2/Smpd3) (modulates the Ca^2+^-dependent secretion of EVs) gene in *mdx* mice led to a decrease in muscle inflammation and overall improved functionality (Matsuzaka et al., 2020). These studies already point toward the multidimensional role of EVs in DMD.

EVs also participate in the physiological regulation of both skeletal and cardiac muscle. The roles of EVs released by cardiac and non-cardiac cells in the heart are difficult to define. Most current studies have not been able to mimic physiologically-relevant environments that can be closely associated with *in vivo* processes (Wagner & Radisic, 2021). On the other hand, in order to maintain the metabolic balance of the body, physiological regulation of organ cross-talk is essential (Wang et al., 2022). As an endocrine organ, skeletal muscle can release a multitude of factors that are absorbed by various tissues to produce regulatory effects (Leal et al., 2018). In addition to these factors, skeletal muscle can also release EVs, especially during physical exercise (Rome et al., 2019). EVs released during exercise are released by skeletal muscle and absorbed by other tissues, leading to intercellular and interorgan communication, effectively altering the metabolism of receptor cells (Guay & Regazzi, 2017). In this sense, skeletal muscle promotes systemic health through the release of EVs after exercise. Physical therapy is a very important intervention method for DMD patients (Case et al., 2018), therefore, the importance of EVs released during exercise is also crucial in pathological settings.

## 4 The role of extracellular vesicles in the Duchenne muscular dystrophy skeletal muscle phenotype

It has become quite well-known that EVs derived from different sources (healthy) can elicit functional improvements in different disease models (Biera et al., 2018) (Leng et al., 2021). Most of this research has already indicated that EVs play a key role in the pathology of brain diseases in particular (Christoforidou et al., 2020) (Meldolesi, 2021). Not much has been revealed about the role of EVs in DMD pathology (mechanism), however, more studies are now focusing on this phenomenon. It is important to note that the paracrine actions displayed by skeletal muscle-released EVs is not exclusive to muscle physiology, they may also have an impact on whole-body homeostasis (Rome et al., 2019). Recently, our laboratory has pointed at the anti-myogenic role of EVs derived from dystrophic mice on C2C12 myoblasts and human mesoangioblasts (Yedigaryan et al., 2022b). As stated previously, the myomiRs represent muscle-oriented miRNAs and likely epitomize the most efficacious molecules found mainly in EVs, and tied to striated muscle physiology. Studies on myomiRs miR-1, miR-133, and miR-206 specifically, have gained tremendous attention in understanding muscle degeneration. Accordingly, we found that miR-206 was upregulated in mainly dystrophic, but also aged mice (Yedigaryan et al., 2022b). Consequently, one of the main targets of miR-206 is the ribosomal binding protein 1, a potent regulator of collagen biosynthesis specifically in fibrogenic recipient cells (Fry et al., 2017). Therefore, the upregulation of this miRNA would induce less extracellular matrix deposition, which would be the optimal setting for muscle remodeling, in particular, hypertrophy. However, in DMD, this could also explain the fibrotic pathogenesis, since satellite cell activity is perturbed and, therefore, the muscle cannot be properly regenerated.

Although not in the context of DMD, a recent study displayed that myoblasts incubated with EVs released from myotubes pre-treated with an oxidative stress-mimicking substance, namely hydrogen peroxide, lead to a significant reduction in the diameter of myotubes and to a stimulation of myoblast proliferation (Guescini et al., 2017). This goes in line with the grave role of oxidative stress involved in the pathogenesis of DMD (Petrillo et al., 2017) (Duelen et al., 2022). It has even been highlighted as being the prime cause for muscle degeneration in DMD (Salam et al., 2007). Therefore, under such diseased conditions, this could potentially be a role for skeletal muscle EVs to induce further oxidative stress.

Metabolically, Aswad et al. revealed that the excessive concentration of palmitate and saturated fatty acids in diet-induced obese mice increases intra-muscular ceramide levels that have been established to regulate EV release from muscle (Aswad et al., 2014). In a later study, it was found that an inhibitor of ceramide synthesis GW4869 (an inhibitor of exosome secretion) was able to improve muscle structure and function in *mdx* mice compared to untreated *mdx* mice (Matsuzaka et al., 2016). This gives more evidence to uphold the concept of deleterious organ cross-talks associated with the circulation of detrimental EV cargos.

**FIGURE 2:**
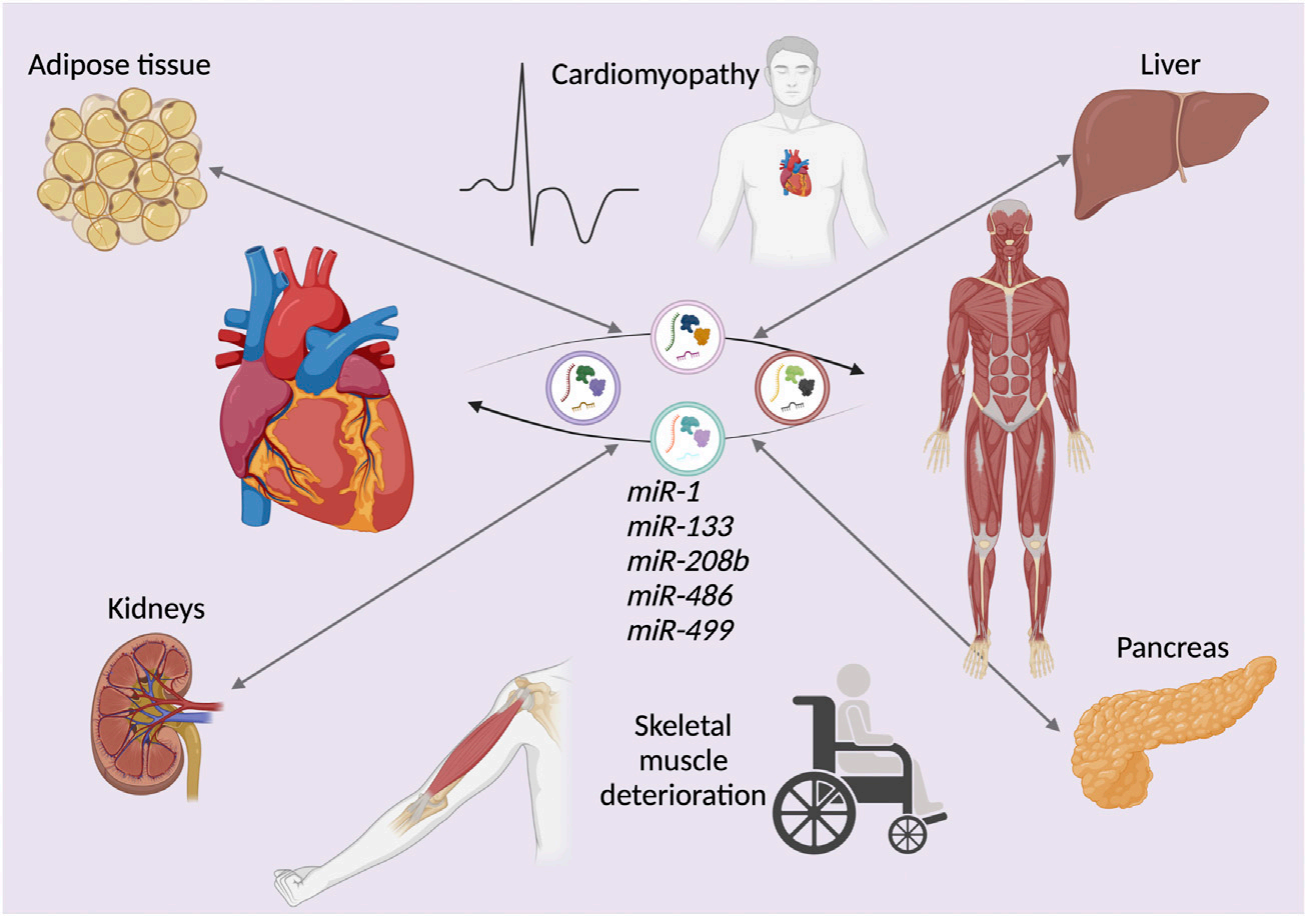
The deleterious effects of extracellular vesicle (EV)-mediated organ cross-talk. The exchange of microRNAs (miRNAs), either downregulated or upregulated in young and elderly Duchenne muscular dystrophy (DMD) patients. The depicted miRNAs represent examples of EV cargos exchanged between not only cardiac and skeletal muscle, but also various other organs and tissues such as kidneys, pancreas, liver, and adipose tissue. This cross-talk exacerbates the DMD phenotype.

Research has shown that fluorescently-labeled skeletal muscle-derived exosomes injected in mice are efficiently taken up by the heart (Aswad et al., 2014). On the other hand, cardiosphere-derived cell (CDC) exosomes have been found to be taken up by skeletal muscle (Aminzadeh et al., 2018). Cardiospheres are self-organized spherical clusters derived from human cardiac stem cells (Messina et al., 2004). These cells can be replated to yield CDCs (Smith et al., 2007). The connection between heart failure and idiosyncrasies in skeletal muscle morphology and biological function has been well documented (Lipkin et al., 1988) (Sullivan et al., 1990). Evidence suggests that myocardium-derived miRNAs released into circulation during heart failure progression may have a negative impact on skeletal muscle (Murach & McCarthy, 2017). This reveals another potential role of EVs released in dystrophic muscles, contributing to progressive muscle degeneration due to the cross-talk between skeletal and cardiac muscle EVs, as progressive cardiomyopathy progression and skeletal muscle dissociation in DMD ostensibly leads to the exchange of deleterious EVs from both organs, escalating disease progression. Both skeletal muscle and cardiac muscle partake in multiple organ cross-talks *via* EVs (Figure 2).

This also may contribute to the idea that therapy (cell or cell-free) targeting both cardiac and skeletal muscles would be of great interest when focusing on the most debilitating manifestations of muscular dystrophies, especially DMD. Recently, our laboratory developed induced pluripotent stem cell (iPSC)-derived mesodermal progenitors through differentiation in a monolayer and chemically-defined media (Breuls et al., 2021). We found that these cells are able to differentiate into the skeletal and cardiac muscle lineages both *in vitro* and *in vivo*. Therefore, these cells may house a secretome that could be beneficial to both striated muscle types.

As stated previously, for now, miRNAs appear to represent the most consequential population of EV cargos. This applies not only to the myomiRs, but also to more ubiquitously expressed miRNAs (Catapano et al., 2020). In animal models of DMD, some miRNAs have been found to be significantly up- or downregulated (Yedigaryan & Sampaolesi, 2021). These miRNAs include miR-199a-5p (upregulated in DMD muscle cells and downregulated in DMD fibroblasts (Zanotti et al., 2018)), involved in the wingless-related integration site (WNT) signaling pathway (targeting numerous myogenic cell proliferation and differentiation regulatory factors) (Alexander et al., 2013), and miR-29, a targeter of AKT Serine/Threonine Kinase 3 (AKT3)/nuclear factor kappa-light-chain-enhancer of activated B cells (NF-kB)/Yin Yang1 (YY1) signaling and fibrotic genes (Wang et al., 2012). Recently, miR-486 has also been revealed to play an essential role in muscle function and is found to be significantly downregulated in DMD (Samani et al., 2022). The loss of this miRNA has been associated with disrupted muscle architecture, reduced myofiber size, and increased cardiac fibrosis, just to name a few. Some miRNAs have been revealed to be exclusively upregulated in DMD *in vitro* and *in vivo* models. These miRNAs include, miR-200c, targeter of forkhead box protein O1 (FOXO1) and endothelial nitric oxide synthase (eNOS), essentially inhibiting myoblast differentiation (D'Agostino et al., 2018), miR-21, targeting phosphatase and tensin homolog (PTEN) and receptor tyrosine kinase (RTK) signaling, ultimately leading to more collagen production (Zanotti et al., 2015), and miR-31, directly targeting Myf5 and dystrophin, leading to satellite cell quiescence and a furthermore contribution to the lack of dystrophin (Young et al., 2016).

miRNAs also represent potentially exceptional biomarkers for DMD (Coenen-Stass et al., 2017). miRNAs are abundant, easily measured using low-cost techniques, stable in properly stored biofluid samples, and the discovery of candidate biomarkers is facile compared to novel protein biomarkers. Not only are most myomiRs great candidates for biomarkers, but additional putative biomarkers have been revealed to be associated with DMD, including: miR-30, miR-22, miR-95, miR-181, miR-193b, and miR-378 (Jeanson-Leh et al., 2014). Jeanson-Leh et al. also found that in the serum of the Golden Retriever muscular dystrophy model there is a class of dysregulated miRNAs, namely members of the delta like non-canonical Notch ligand 1-iodothyronine deiodinase 3 (*DLK-DIO3*) locus. This locus appears to have an involvement in muscle pathophysiology (Benetatos et al., 2013).

The specific types of surface antigens on EVs have also been considered in the realm of biomarker adaptation for DMD. As an example, Matsuzaka et al. found that an increase in the levels of muscle-abundant miRNAs appears to be tied to CD63 antigen levels on EVs (Matsuzaka et al., 2016). They found that CD63-and major histocompatibility complex (MHC) II-associated EVs collected from the serum of DMD patients were significantly enriched in myomiRs compared to healthy controls, especially miR-1, miR-133a, and miR-206 for CD63-associated EVs and miR-1 and miR-133a for MHC II-associated EVs. Additionally, in *mdx* mice, it was found that levels of miR-133a and miR-206 were significantly increased in CD81-, flotillin-1-, and MHC II-associated EVs, and flotillin-1- and MHC II-coupled EVs, respectively, compared to wild type mice.

Overall, skeletal muscle-released EVs from DMD models must still be additionally explored in order to truly understand their role in DMD pathophysiology and to start the establishment of further therapeutic strategies involving EVs.

## 5 Cardiac involvement of extracellular vesicles in Duchenne muscular dystrophy

It has become apparent that there is a wealth of molecular species released by cardiac muscle under both physiological and pathological circumstances (Valadi et al., 2007) (Pironti et al., 2015) (Gao et al., 2020). These molecules are said to employ paracrine and/or endocrine effects (Gabisonia et al., 2022). Contrariwise, humoral factors produced by other organs, such as adipose tissue (Crewe et al., 2021), liver (Zardi et al., 2010), kidney (Gao et al., 2020), or skeletal muscle (Murach & McCarthy, 2017), may impact the function of normal and diseased hearts. EVs represent one of the main means of delivering potent molecules to and from the heart. Furthermore, intracardiac cell-to-cell communication has also come to the forefront of understanding the paracrine and endocrine effects of EVs in the heart (Saheera et al., 2021) (Nguyen et al., 2021). It has been found that cardiomyocytes represent only approximately 30% of cardiac cells, therefore, they must have a means of communicating with neighboring endothelial cells, fibroblasts, smooth muscle cells, macrophages, and pericytes (Nag, 1980) (Banerjee et al., 2007). The proper communication between these cell types ensures the homeostatic state of the heart (Perbellini et al., 2018). A multitude of different proteins and nucleic acids have been identified in cardiomyocyte-released exosomes (Malik et al., 2013). Some examples include inflammatory factors such as interleukin-6 (IL-6) (Datta et al., 2017), IL-1β (Yu et al., 2019), tumor necrosis factor-α (TNF-α) (Yu et al., 2012), heat shock proteins such as heat-shock protein 20 (Hsp20) (Zhang et al., 2012), metabolic transporters such as glucose transporter type 4 (GLUT4) and type 1 (GLUT1) (Garcia et al., 2016), as well as enzymes such as lactate dehydrogenase (LDH), and proteins that play a major role in the cardiac development signaling pathway, such as Wnt-binding proteins (Korkut et al., 2009). As stated previously, nucleic acids, especially a wide variety of miRNAs are also present in cardiac EVs (Waldenström et al., 2012) (Wang et al., 2014) (Li et al., 2021). The specific cargo carried by these EVs is strongly tied to their biogenesis and by the different stimuli influencing the parent cells (Malik et al., 2013).

Since cardiomyopathy is one of the leading causes of early mortality in DMD patients, it is crucial to understand the molecular pathology leading to cardiac dysfunction. Gartz et al. demonstrated that short-term exposure to exosomes secreted by dystrophin-deficient iPSC-derived cardiomyocytes (Dys-iCM) had the same protective effect against stress-induced injury on Dys-iCMs as did wild type exosomes (Gartz et al., 2018). They revealed that the protective pathways stimulated in this process included p38 mitogen-activated protein kinase (p38 MAPK) and extracellular signal-regulated protein kinase 1/2 (ERK1/2). However, since this did not mimic physiological *in vivo* conditions where cells are exposed to secreted exosomes continuously, Gartz et al. made a follow-up study to see if long-term exposure to exosomes may represent physiological conditions better (Gartz et al., 2020). In this follow-up study, Gartz et al. tested the effects of DMD cardiac exosomes (acquired from iPSC-derived DMD cardiomyocytes) on iPSC-derived DMD cardiomyocytes, after long-term exposure (Gartz et al., 2020). They confirmed that DMD cardiomyocytes were more vulnerable to stress (higher levels of reactive oxygen species), exhibited a decrease in mitochondrial membrane potential, and experienced an increase in the level of cell death. Long-term exposure of these cardiomyocytes to non-affected exosomes revealed to be protective, however, exposure to DMD cardiac exosomes resulted in no protective effects, rather an increase in the vulnerability to stress of DMD cardiomyocytes. Furthermore, they found that the miRNA cargo was significantly implicated in the pathological effects of DMD cardiac exosomes. In particular, the miRNA cargo housed miRNAs that regulate adverse pathways such as tumor protein P53 (p53) and transforming growth factor-β (TGF-β) (Shirokova & Niggli, 2013) (Lin et al., 2015). Lastly, it was also revealed that exosome inhibition mitigated pathological events tied to DMD cardiomyopathy in an *mdx* mouse model. The most recent follow-up study surfaced where the authors tried to identify the exact mechanism by which DMD cardiac exosomes are able to alter crucial stress-responsive genes in recipient cells (Gartz et al., 2021). They revealed that iPSC-derived DMD cardiomyocyte exosomes contain a heavily-altered miRNA profile compared to healthy controls. In particular, miR-339-5p was upregulated in DMD cardiomyocytes, DMD cardiac exosomes, as well as in mouse cardiac tissue. Some of the important targets of this miRNA are mouse double minute 2 homolog (MDM2), glycogen synthase kinase-3 alpha (GSK3A), and mitogen-activated protein kinase kinase 3 (MAP2K3), genes that are important in stress-responsive signaling pathways (Betel et al., 2008) (Zhang et al., 2014) (Jansson et al., 2015) (Agarwal et al., 2015). Therefore, the upregulation of this miRNA diminishes the ability of DMD cardiomyocytes to protect against stress injury. In line with miRNA deregulation, the myomiRs also appear to be abnormally expressed in the heart of DMD models (Lee et al., 2022). A few examples include, miR-1, involved in the electrical remodeling of the heart and arrhythmia induction if dysregulated (Yang et al., 2007), miR-133, which in case of downregulation causes cardiac hypertrophy (Carè et al., 2007), and the miR-208 family that strongly contributes to cardiac hypertrophy and arrhythmias (Callis et al., 2009).

Other studies have focused more on the potential positive/negative effects of EVs derived from healthy/diseased cells and/or tissues. Research has shown that exosomes isolated from healthy wild-type CDCs *in vitro* are able to improve both cardiac function and skeletal muscle myopathy (Aminzadeh et al., 2018) (Rogers et al., 2019). This effect was the result of a decrease in inflammation and oxidative stress, as well as improved mitochondrial function. Additionally, miR-148a was identified as a potential facilitator of enhanced full-length dystrophin protein synthesis. Another study revealed that exosomes collected from progenitor or stem cells *in vitro*, are able to improve cardiac function *in vivo*, after injury (Lai et al., 2010). In other cardiac disorders, namely cardiac overload or diabetic cardiomyopathy, it was found that exosomes hamper cardiomyocyte metabolism (Halkein et al., 2013) (Bang et al., 2014) or induce cardiac hypertrophy (Bang et al., 2014) through the delivery of an abundance of pathogenic miRNAs. More recently, it was discovered that exosomes isolated from DMD muscle-derived fibroblasts stimulate fibrosis, mainly mediated by exosomal miR-199-5p (Zanotti et al., 2018). With regard to three dimensional (3D) cardiac models, recently, our laboratory unveiled that cardiac organoids derived from DMD iPSCs display DMD-like cardiomyopathy and disease progression phenotypes in long-term cultures compared to clustered regularly interspaced short palindromic repeats (CRISPR)/(CRISPR/Cas9) mutation-corrected isogenic controls (Marini et al., 2022). It was revealed that in the DMD cardiac organoid dysregulated gene network, three miRNAs were found to play crucial roles. These miRNAs were hsa-mir-335-5p, hsa-mir-124-3p, and hsa-mir-26b-5p and further studies are in progress to determine their gene targets and potential roles in DMD cardiac degeneration. miR-335-5p is said to play a critical role in cardiomyocyte apoptosis, calcium level increases, and cardiac hypertrophy in right ventricular remodeling due to pulmonary arterial hypertension (Ma et al., 2022). miR-124-3p has been described to promote cardiac fibroblast activation and proliferation in patients with atrial fibrillation (Zhu et al., 2022). Lastly, miR-26b-5p has been found to be highly expressed in phenylephrine-induced cardiac hypertrophy (Tang et al., 2020).

As with skeletal muscle, miRNAs are also becoming strong candidate biomarkers for DMD cardiomyopathy progression. Although less pronounced than skeletal muscle biomarkers, some miRNAs have been found to be tied to specific muscular dystrophy cardiac events (Becker et al., 2016). As an example, Becker et al. revealed that miR-222, miR-26a, and miR-378a-5p, are significantly upregulated in patients with proof of myocardial scarring compared to those without (Becker et al., 2016). A more recent study has revealed that serum levels of miR-1, miR-133a, miR-133b, and miR-499 were significantly higher in DMD patients with cardiac involvement compared to the no cardiac involvement group (Meng et al., 2022). It has come to attention that the lipid signature of EVs may also discriminate between diseased and control conditions. One such example was evidenced by Burrello et al., where they identified a significant shift in the amount, as well as the diversity of sphingolipid species in EVs derived from ST-segment-elevation myocardial infarction patients *versus* controls (Burrello et al., 2020).

Although research revealing the role of EVs in DMD cardiomyopathy is quite novel, there are some promising results that confirm the potential pathophysiological roles of diseased EVs. Indeed, as documented in the next section, some clinical trials reveal a central role of miRNAs in the positive action of cell secretome-mediated therapy. Please see Table 1 for an overview of important miRNAs described in the text.

**TABLE 1:** An overview of dysregulated microRNAs (miRNAs) in Duchenne muscular dystrophy (DMD) described in the text. The columns represent the miRNAs, up- or downregulated in skeletal and/or cardiac muscle, and their potential involvement in DMD due to dysregulation (*in vivo* and/or *in vitro*).

microRNAs	Sk—skeletal muscle, C—cardiac muscle Up- or downregulated (specified in text)	Study type and potential involvement in DMD due to dysregulation
miR-1	C: Up	*In vivo*; Arrhythmogenesis
miR-133a	C: Down	*In vivo* and *in vitro*; Cardiac hypertrophy
miR-206	Sk: Up	*In vitro*; Fibrosis
miR-199-5p	Sk: Up, C: Up	*In vivo* and *in vitro*; Fibrosis
miR-29	Sk: Down	*In vivo* and *in vitro*; Increased muscle fibrogenesis
miR-486	Sk: Down, C: Down	*In vivo*; Disrupted muscle architecture, reduced myofiber size, and increased cardiac fibrosis
miR-200c	Sk: Up	*In vivo* and *in vitro*; Reduced myofiber size
miR-21	Sk: Up	*In vivo* and *in vitro*; Fibrosis
miR-31	Sk: Up	*In vivo* and *in vitro*; Reduced myofiber size and disrupted muscle architecture
miR-339-5p	C: Up	*In vivo and in vitro*; Impairment of protection from stress injury, cell death
miR-208	C: Up	*In vivo*; Cardiac hypertrophy
miR-148a	C: Down	*In vivo* and *in vitro*; Facilitator of dystrophin synthesis
miR-335-5p	C:*	*In vitro*; DMD cardiac organoid dysregulation, cardiomyocyte apoptosis, increase in calcium levels, and cardiac hypertrophy
miR-124-3p	C:*	*In vitro*; DMD cardiac organoid dysregulation, cardiac fibrosis
miR-26b-5p	C:*	*In vitro*; DMD cardiac organoid dysregulation, cardiac hypertrophy

*These miRNAs are said to be dysregulated in DMD cardiac organoids (not specified yet if up- or downregulated).

## 6 From the bench to the bedside: Extracellular vesicles in clinical development

The use of human embryonic stem cells (hESCs) or hESC-derived cells in the field of cardiac regeneration has progressed towards a clinical trial (Menasché et al., 2018). Menasché et al. conducted a trial for the use of hESC-derived cardiovascular progenitors (embedded in a fibrin patch) on patients with severe ischemic left ventricular dysfunction. They demonstrated the feasibility of deriving these cells in a clinical-grade manner, as well as outlined the short- and medium-term safety of these cells. Although interpreted cautiously, the authors did detect improved contractility of the grafted segments. This improvement was hypothesized to be due to the secretion of biomolecules manifesting host-linked reparative mechanisms (Garbern & Lee, 2013). These biomolecules were found to be largely present in EVs. Furthermore, it was previously shown that the cardioprotective effects of the hESC-derived cardiac progenitors could be recapitulated by the sole administration of their released EVs (Kervadec et al., 2016). The authors assessed the content of these EVs and found that miR-302 appeared to be predominantly expressed. This miRNA is tied to cardiomyocyte proliferation stimulation (Tao et al., 2015). This revelation has caused a further understanding and a potential shift towards viewing hESC- or iPSC-derived progenitors as vital producers of a secretome able to induce a paracrine mechanism of action, explaining the long-term action of ESC derivatives in spite of their scarce long-standing engraftment potential (Menasché, 2022). Therefore, the secretome may be further considered as the “active substance”, that includes not only cytokines, chemokines, growth factors, RNAs, and proteins, but also extracellular vesicles. This ongoing study also exudes a burgeoning high potency when it comes to a multitude of other cardiac diseases, including DMD-induced cardiomyopathy.

Early work reported by Aminzadeh et al. (Aminzadeh et al., 2014) (Aminzadeh et al., 2015) motivated the Halt cardiomyOPathy progrEssion in Duchenne (HOPE-Duchenne) clinical trial (Ascheim & Jefferies, 2016), which is now entering phase 3 (McDonald et al., 2022). Aminzadeh et al. unveiled the striking effect of CDCs in *mdx* mice. Although initially the main goal was to ameliorate some of the pathophysiological consequences of DMD in the heart, the authors later revealed the affinity of CDCs to also restore skeletal muscle function (Aminzadeh et al., 2018). This uncovered the major systemic benefits of CDC injection into the heart. CDCs are said to induce their beneficial effects through the secretion of exosomes. These exosomes may play a multitude of different roles such as targeting macrophages and altering them into a healing rather than pro-inflammatory state (de Couto et al., 2015), as well as targeting fibroblasts and ultimately having an antifibrotic effect (Tseliou et al., 2015). Among the cargo of CDC-derived exosomes, miRNAs (miR-146a and miR-181b) and Y RNA fragments are the known bioactive components (Cambier et al., 2017) (de Couto et al., 2017) (Marbán, 2018) (McDonald, 2022). miR-146b has been shown to suppress ischemia/reperfusion injury by targeting interleukin-1 receptor-associated kinase 1 (IRAK) and tumor necrosis factor receptor (TNFR)-associated factor 6 (TRAF6) (Cheng et al., 2013) (Ibrahim et al., 2014). These pro-inflammatory cytokines are involved in the toll-like receptor (TLR) signaling pathway (Wang et al., 2013). This miRNA was shown to also suppress nicotinamide adenine dinucleotide phosphate oxidase four (NOX4) and SMAD family member 4 (SMAD4), which are involved in oxidative stress induction and the TGF-β profibrotic pathway, respectively (Infanger et al., 2010) (Vasa-Nicotera et al., 2011) (Liu et al., 2012). It has been found that miR-181b may be involved in the cardioprotective effect of CDC-derived exosomes by targeting protein kinase C-delta (PKC-δ) within distinctive macrophages, to induce a marked polarization state (de Couto et al., 2017). Cambier et al. revealed that a fragment of Y RNA, highly enriched in CDC-derived EVs, enhances IL-10 protein secretion by altering *Il10* gene expression (Cambier et al., 2017). IL-10 is a well-known cardioprotective cytokine, particularly in myocardial ischemia/reperfusion injury (Yang et al., 2000).

Phase 1 of the HOPE clinical study revealed that CDCs appeared to display an acceptable level of safety and were able to preserve upper limb and cardiac function after one-time delivery into the coronary arteries of DMD patients (Ascheim & Jefferies, 2016) (McDonald et al., 2022). Phase 2 of the HOPE clinical trial (HOPE-2) examined the safety and efficacy of sequential intravenous injections of human CDCs in patients with late-stage DMD (McDonald, 2018). This trial disclosed that CDCs are safe and efficacious with regard to reducing deterioration of upper limb function in late-stage DMD patients. Cardiac functional and structural benefits were also observed compared to the placebo group. Phase 3 of the HOPE clinical trial (HOPE-3) is currently recruiting participants and will entail testing the CDCs in a large number of patients, compared to phase 2 (McDonald et al., 2022). Therefore, the HOPE clinical trial represents a very promising therapeutic approach for DMD patients, as it continues on to the third phase of testing.

It is imperative to develop a large-scale production of EVs as a medicinal product in line with current good manufacturing processes (GMP) guidelines. Such methods are currently being studied and developed. Overcoming limitations such as contaminants, xeno-free reagents *in lieu* of animal-derived ones, and scalable and reliable isolation methods are some of the important aspects of establishing ready-to-use EV products (Andriolo et al., 2018). It is also critical that the robustness and the reproducibility be measurable with reliable and optimized analytical methods in order to follow GMP guidelines.

Overall, the future of research in the direction of understanding the importance of the cell secretome is gaining momentum and more revelations with regard to this topic will surely be made.

## 7 Conclusion and potential therapeutic applications

**FIGURE 3:**
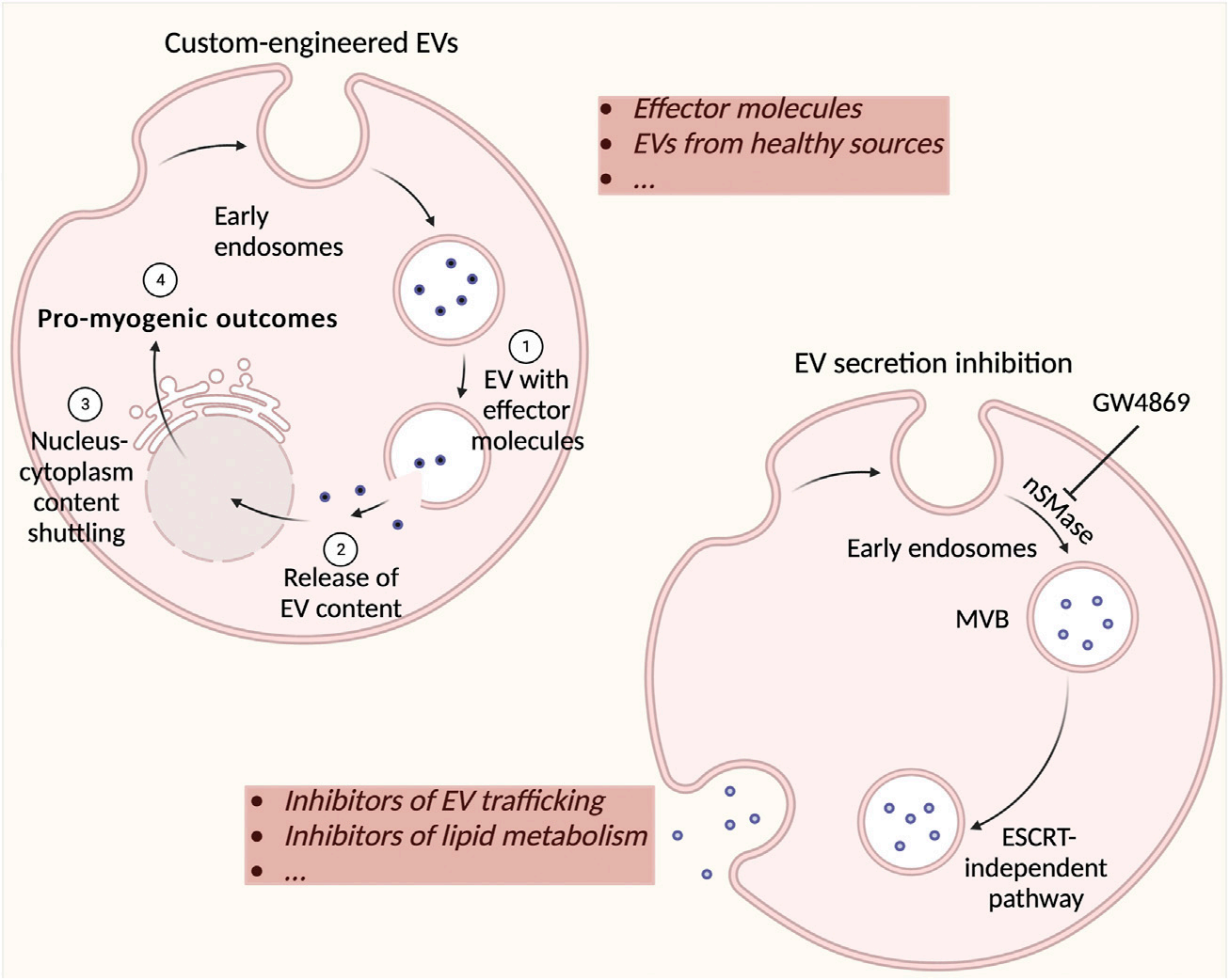
Possible strategies for using extracellular vesicles (EVs) as therapeutic agents. Custom cargos may be implemented in specific EVs (such as derived from healthy muscle) and potentially used as a Duchenne muscular dystrophy (DMD) therapeutic. Effector molecules may include nucleic acids, lipids, proteins, adeno-associated viruses (for gene therapy), and propeptides with limited efficacy via direct administration. The inhibition of EV formation and secretion is a new strategy for stopping the effect of detrimental EVs in DMD. Most commonly used compounds include inhibitors of EV trafficking such as calpeptin, manumycin A, Y27632, and inhibitors of lipid metabolism such as imipramine, pantethine, and GW4869. These inhibitors may either act on the endosomal sorting complexes required for transport machinery (ESCRT)-dependent (e.g. manumycin A) or –independent pathway (e.g. GW4869). There are a multitude of other drugs that have been investigated for their ability to inhibit EV secretion such as U0126, clopidogrel, indomethacin, and chloramidine. These strategies represent potentially highly efficacious therapeutics for DMD treatment.

In retrospect, EVs represent a field of study that must be explored a lot more in depth. While recent studies have already established that EVs are very important mediators in cell-to-cell communication, it remains to be further seen exactly how they contribute to healthy compared to diseased physiology. There is an abundant amount of evidence suggesting that DMD EVs induce deleterious effects, so it may be beneficial to understand which molecules are responsible for the adverse effects and to explore the possibility of counteracting the release of these molecules from EVs. Nowadays, the focus of EV-related potential therapies lies with the delivery of either EVs derived from healthy sources (Aminzadeh et al., 2018) (Yedigaryan et al., 2022a) or custom-cargo-engineered EVs that can efficiently deliver molecules to recipient cells (Matsuzaka et al., 2022). The inhibition of EV release represents a paradigm shift in the manner of treatment for diseases (Gartz et al., 2020) (Catalano & O'Driscoll, 2019) (Figure 3). However, compounds used to inhibit EV formation and/or release must be scrutinized closely for their off-target effects and under these circumstances, investigated for their influence on healthy cells. In the context of DMD, it appears that one of the most efficacious molecules in EVs are miRNAs. This also asserts a potential therapeutic strategy, as antagomiRs (anti-miRs), chemically engineered oligonucleotides intended for silencing endogenous miRNAs, may be utilized to block harmful miRNAs. On the other hand, miRNA mimics, chemically-designed double-stranded RNA molecules used to imitate endogenous miRNAs, may be used to enrich tissues with advantageous miRNAs (Rotini et al., 2018). These anti-miRs could potentially also be encapsulated in healthy EVs and delivered to patients. Although these ideas all seem to represent a highly compelling argument for their use in DMD therapy, much research and trials must be devised to understand their feasibility, safety, and final effectiveness (Villa et al., 2019). However, nano-therapy is becoming increasingly widespread and there will most likely come a time when exploratory cell therapy takes a backseat to cell-free therapeutic approaches.

## References

[B1] Aartsma-Rus A. Straub V. Hemmings R. Haas M. Schlosser-Weber G. Stoyanova-Beninska V. (2017). Development of exon skipping therapies for Duchenne muscular dystrophy: A critical review and a perspective on the outstanding issues. Nucleic Acid. Ther. 27 (5), 251–259. 28796573 10.1089/nat.2017.0682 PMC5649120

[B2] Agarwal V. Bell G. W. Nam J.-W. Bartel D. P. (2015). Predicting effective microRNA target sites in mammalian mRNAs. eLife 4, e05005. 26267216 10.7554/eLife.05005 PMC4532895

[B3] Alexander M. S. Kawahara G. Motohashi N. Casar J. C. Eisenberg I. Myers J. A. (2013). MicroRNA-199a is induced in dystrophic muscle and affects WNT signaling, cell proliferation, and myogenic differentiation. Cell Death Differ. 20 (9), 1194–1208. 10.1038/cdd.2013.62 23764775 PMC3741500

[B4] Aminzadeh M. A. Tobin R. Smith R. Marbán L. Marbán E. (2014). Heart-derived cell therapy for Duchenne cardiomyopathy: Cardiosphere-derived cells and their exosomes improve function, restore mitochondrial integrity and reverse degenerative changes in the hearts of mdx mice. Circulation Res. 115, e90.

[B5] Aminzadeh M. Durvasula P. Tobin R. Guan X. Andres A. Taylor D. (2015). Exosome-mediated reversal of Duchenne cardiomyopathy. Circulation 132, A16015.

[B6] Aminzadeh M. Rogers R. Fournier M. Tobin R. Guan X. Childers M. (2018). Exosome-mediated benefits of cell therapy in mouse and human models of Duchenne muscular dystrophy. Stem Cell Rep. 10 (3), 942–955. 10.1016/j.stemcr.2018.01.023 PMC5918344 29478899

[B7] Andriolo G. Provasi E. Lo Cicero V. Brambilla A. Soncin S. Torre T. (2018). Exosomes from human cardiac progenitor cells for therapeutic applications: Development of a GMP-grade manufacturing method. Front. Physiology 9, 1169. 10.3389/fphys.2018.01169 PMC6117231 30197601

[B8] Annibalini G. Contarelli S. Lucertini F. Guescini M. Maggio S. Ceccaroli P. (2019). Muscle and systemic molecular responses to a single flywheel based iso-inertial training session in resistance-trained men. Front. Physiology 10, 554. 10.3389/fphys.2019.00554 PMC6521220 31143128

[B9] Aravind S. Ashley B. Mannan A. Ganapathy A. Ramesh K. Ramachandran A. (2019). Targeted sequencing of the DMD locus: A comprehensive diagnostic tool for all mutations. Indian J. Med. Res. 150 (3), 282–289. 10.4103/ijmr.IJMR_290_18 31719299 PMC6886143

[B10] Arpke R. W. Darabi R. Mader T. L. Zhang Y. Toyama A. Lonetree C.-l. (2013). A new immuno-dystrophin-deficient model, the NSG-mdx4Cv mouse, provides evidence for functional improvement following allogeneic satellite cell transplantation. Stem Cells 31 (8), 1611–1620. 10.1002/stem.1402 23606600 PMC3767774

[B11] Ascheim D. Jefferies J. (2016). A randomized, open-label study of the safety and efficacy of multi-vessel intracoronary delivery of allogeneic cardiosphere-derived cells in patients with cardiomyopathy secondary to Duchenne muscular dystrophy. Available at: https://clinicaltrials.gov/ct2/show/NCT02485938 .

[B12] Aswad H. Forterre A. Wiklander O. P. Vial G. Santy-Berger E. Jalabert A. (2014). Exosomes participate in the alteration of muscle homeostasis during lipid-induced insulin resistance in mice. Diabetologia 57, 2155–2164. 10.1007/s00125-014-3337-2 25073444 PMC4153976

[B13] Atkin-Smith G. K. Tixeira R. Paone S. Mathivanan S. Collins C. Liem M. (2015). A novel mechanism of generating extracellular vesicles during apoptosis via a beads-on-a-string membrane structure. Nat. Commun. 6, 7439. 10.1038/ncomms8439 26074490 PMC4490561

[B14] Banerjee I. Fuseler J. W. Price R. L. Borg T. K. Baudino T. A. (2007). Determination of cell types and numbers during cardiac development in the neonatal and adult rat and mouse. Am. J. Physiology Heart Circulatory Physiology 293 (3), H1883–H1891. 10.1152/ajpheart.00514.2007 17604329

[B15] Bang C. Batkai S. Dangwal S. Gupta S. K. Foinquinos A. Holzmann A. (2014). Cardiac fibroblast-derived microRNA passenger strand-enriched exosomes mediate cardiomyocyte hypertrophy. J. Clin. Investigation 124 (5), 2136–2146. 10.1172/JCI70577 PMC4001534 24743145

[B16] Becker S. Florian A. Patrascu A. Rösch S. Waltenberger J. Sechtem U. (2016). Identification of cardiomyopathy associated circulating miRNA biomarkers in patients with muscular dystrophy using a complementary cardiovascular magnetic resonance and plasma profiling approach. J. Cardiovasc. Magnetic Reson. 18, 25. 10.1186/s12968-016-0244-3 PMC4858897 27150296

[B17] Benetatos L. Hatzimichael E. Londin E. Vartholomatos G. Loher P. Rigoutsos I. (2013). The microRNAs within the DLK1-DIO3 genomic region: Involvement in disease pathogenesis. Cell. Mol. Life Sci. 70, 795–814. 10.1007/s00018-012-1080-8 22825660 PMC11114045

[B18] Bentzinger C. F. Wang Y. X. Rudnicki M. A. (2012). Building muscle: Molecular regulation of myogenesis. Cold Spring Harb. Perspect. Biol. 4, a008342. 10.1101/cshperspect.a008342 22300977 PMC3281568

[B19] Betel D. Wilson M. Gabow A. Marks D. A. Sander C. (2008). The microRNA.org resource: Targets and expression. Nucleic Acids Res. 36 (1), D149–D153. 10.1093/nar/gkm995 18158296 PMC2238905

[B20] Biera A. Berensteina P. Kronfelda N. Morgoulis D. Ziv-Av A. Hodaya G. (2018). Placenta-derived mesenchymal stromal cells and their exosomes exert therapeutic effects in Duchenne muscular dystrophy. Biomaterials 174, 67–78. 10.1016/j.biomaterials.2018.04.055 29783118

[B21] Bonilla E. Samitt C. E. Miranda A. F. Hays A. P. Salviati G. DiMauro S. (1988). Duchenne muscular dystrophy: Deficiency of dystrophin at the muscle cell surface. Cell 54 (4), 447–452. 10.1016/0092-8674(88)90065-7 3042151

[B22] Breuls N. Giarratana N. Yedigaryan L. Garrido G. M. Carai P. Heymans S. (2021). Valproic acid stimulates myogenesis in pluripotent stem cell-derived mesodermal progenitors in a NOTCH-dependent manner. Cell Death Dis. 12, 677. 10.1038/s41419-021-03936-w 34226515 PMC8257578

[B23] Burrello J. Biemmi V. Dei Cas M. Amongero M. Bolis S. Lazzarini E. (2020). Sphingolipid composition of circulating extracellular vesicles after myocardial ischemia. Sci. Rep. 10, 16182. 10.1038/s41598-020-73411-7 32999414 PMC7527456

[B24] Callis E, T. Pandya K. Seok H. Y. Tang R.-H. Tatsuguchi M. Huang Z. P. (2009). MicroRNA-208a is a regulator of cardiac hypertrophy and conduction in mice. J. Clin. Investigation 119 (9), 2772–2786. 10.1172/JCI36154 PMC2735902 19726871

[B25] Cambier L. de Couto G. Ibrahim A. Echavez A. K. Valle J. Liu W. (2017). Y RNA fragment in extracellular vesicles confers cardioprotection via modulation of IL-10 expression and secretion. EMBO Mol. Med. 9 (3), 337–352. 10.15252/emmm.201606924 28167565 PMC5331234

[B26] Carè A. Catalucci D. Bonci D. Addario A. Gallo P. Bang M.-L. (2007). MicroRNA-133 controls cardiac hypertrophy. Nat. Med. 13, 613–618. 10.1038/nm1582 17468766

[B27] Case L. E. Apkon S. D. Gulyas A. Juel L. Matthews D. Newton R. A. (2018). Rehabilitation management of the patient with Duchenne muscular dystrophy. Pediatrics 142 (2), S17–S33. 10.1542/peds.2018-0333D 30275246

[B28] Catalano M. O'Driscoll L. (2019). Inhibiting extracellular vesicles formation and release: A review of EV inhibitors. J. Extracell. Vesicles 9 (1), 1703244. 10.1080/20013078.2019.1703244 32002167 PMC6968539

[B29] Catapano F. Scaglioni D. Maresh K. Ala P. Domingos J. Selby V. (2020). Novel free-circulating and extracellular vesicle-derived miRNAs dysregulated in Duchenne muscular dystrophy. Epigenomics 12 (21), 1899–1915. 10.2217/epi-2020-0052 33215544

[B30] Chamberlain J. R. Chamberlain J. S. (2017). Progress toward gene therapy for Duchenne muscular dystrophy. Mol. Ther. J. Am. Soc. Gene Ther. 25 (5), 1125–1131. 10.1016/j.ymthe.2017.02.019 PMC5417844 28416280

[B31] Chemello F. Wang Z. Li H. R M. J. Liu N. Bassel-Duby R. (2020). Degenerative and regenerative pathways underlying Duchenne muscular dystrophy revealed by single-nucleus RNA sequencing. Proc. Natl. Acad. Sci. U. S. A. 117 (47), 29691–29701. 10.1073/pnas.2018391117 33148801 PMC7703557

[B32] Chen J.-F. Mandel E. M. Thomson J. M. Wu Q. Callis T. E. Hammond S. M. (2006). The role of microRNA-1 and microRNA-133 in skeletal muscle proliferation and differentiation. Nat. Genet. 38 (2), 228–233. 10.1038/ng1725 16380711 PMC2538576

[B33] Cheng H. S. Sivachandran N. Lau A. Boudreau E. Zhao J. L. Baltimore D. (2013). MicroRNA-146 represses endothelial activation by inhibiting pro-inflammatory pathways. EMBO Mol. Med. 5 (7), 1017–1034. 10.1002/emmm.201202318 23733368 PMC3721471

[B34] Christoforidou E. Joilin G. Hafezparast M. (2020). Potential of activated microglia as a source of dysregulated extracellular microRNAs contributing to neurodegeneration in amyotrophic lateral sclerosis. J. Neuroinflammation 17 (1), 135. 10.1186/s12974-020-01822-4 32345319 PMC7187511

[B35] Coenen-Stass A. Betts C. Lee Y. Mäger I. Turunen M. Andaloussi S. (2016). Selective release of muscle-specific, extracellular microRNAs during myogenic differentiation. Hum. Mol. Genet. 25 (18), 3960–3974. 10.1093/hmg/ddw237 27466195 PMC5291232

[B36] Coenen-Stass A. M. Wood M. J. Roberts T. C. (2017). Biomarker potential of extracellular miRNAs in Duchenne muscular dystrophy. Trends Mol. Med. 23 (11), 989–1001. 10.1016/j.molmed.2017.09.002 28988850

[B37] Crewe C. Funcke J.-B. Li S. Joffin N. Gliniak C. M. Ghaben A. L. (2021). Extracellular vesicle-based interorgan transport of mitochondria from energetically stressed adipocytes. Cell Metab. 33 (9), 1853–1868. 10.1016/j.cmet.2021.08.002 34418352 PMC8429176

[B38] Crisafulli S. Sultana J. Andrea F. Salvo F. Messina S. Trifirò G. (2020). Global epidemiology of Duchenne muscular dystrophy: An updated systematic review and meta-analysis. Orphanet J. Rare Dis. 15 (1), 141. 10.1186/s13023-020-01430-8 32503598 PMC7275323

[B39] D'Agostino M. Torcinaro A. Madaro L. Marchetti L. Sileno S. Beji S. (2018). Role of miR-200c in myogenic differentiation impairment via p66Shc: Implication in skeletal muscle regeneration of dystrophic mdx mice. Oxidative Med. Cell. Longev. 2018, 4814696. 10.1155/2018/4814696 PMC5831318 29636844

[B40] Datta R. Bansal T. Rana S. Datta K. Chaudhuri R. D. Chawla-Sarkar M. (2017). Myocyte-derived Hsp90 modulates collagen upregulation via biphasic activation of STAT-3 in fibroblasts during cardiac hypertrophy. Mol. Cell. Biol. 37 (6), e00611–e00616. 10.1128/MCB.00611-16 28031326 PMC5335508

[B41] de Couto G. Gallet R. Cambier L. Jaghatspanyan E. Makkar N. Dawkins J. F. (2017). Exosomal MicroRNA transfer into macrophages mediates cellular postconditioning. Circulation 136 (2), 200–214. 10.1161/CIRCULATIONAHA.116.024590 28411247 PMC5505791

[B42] de Couto G. Liu W. Tseliou E. Sun B. Makkar N. Kanazawa H. (2015). Macrophages mediate cardioprotective cellular postconditioning in acute myocardial infarction. J. Clin. Investigation 125 (8), 3147–3162. 10.1172/JCI81321 PMC4563759 26214527

[B43] Duelen R. Costamagna D. Gilbert G. De Waele L. Goemans N. Desloovere K. (2022). Human iPSC model reveals a central role for NOX4 and oxidative stress in Duchenne cardiomyopathy. Stem Cell Rep. 17 (2), 352–368. 10.1016/j.stemcr.2021.12.019 PMC8828550 35090586

[B44] Dumont N. A. Wang Y. X. von Maltzahn J. Pasut A. Bentzinger C. F. Brun C. E. (2015). Dystrophin expression in muscle stem cells regulates their polarity and asymmetric division. Nat. Med. 21, 1455–1463. 10.1038/nm.3990 26569381 PMC4839960

[B45] Emery A. E. (2002). THe muscular dystrophies. Lancet 359 (9307), 687–695. 10.1016/S0140-6736(02)07815-7 11879882

[B46] Fry C. S. Kirby T. J. Kosmac K. McCarthy J. J. Peterson C. A. (2017). Myogenic progenitor cells control extracellular matrix production by fibroblasts during skeletal muscle hypertrophy. Cell Stem Cell 20 (1), 56–69. 10.1016/j.stem.2016.09.010 27840022 PMC5218963

[B47] Gabisonia K. Khan M. Recchia F. A. (2022). Extracellular vesicle-mediated bidirectional communication between heart and other organs. Am. J. Physiology Heart Circulatory Physiology 322 (5), H769–H784. 10.1152/ajpheart.00659.2021 PMC8993522 35179973

[B48] Gao L. Mei S. Zhang S. Qin Q. Li H. Liao Y. (2020). Cardio-renal exosomes in myocardial infarction serum regulate proangiogenic paracrine signaling in adipose mesenchymal stem cells. Theranostics 10 (3), 1060–1073. 10.7150/thno.37678 31938051 PMC6956822

[B49] Garbern J. C. Lee R. T. (2013). Cardiac stem cell therapy and the promise of heart regeneration. Cell Stem Cell 12 (6), 689–698. 10.1016/j.stem.2013.05.008 23746978 PMC3756309

[B50] Garcia N. A. Moncayo-Arlandi J. Sepulveda P. Diez-Juan A. (2016). Cardiomyocyte exosomes regulate glycolytic flux in endothelium by direct transfer of GLUT transporters and glycolytic enzymes. Cardiovasc. Res. 109 (3), 397–408. 10.1093/cvr/cvv260 26609058

[B51] Gartz M. Beatka M. Prom M. J. Strande J. L. Lawlor M. W. (2021). Cardiomyocyte-produced miR-339-5p mediates pathology in Duchenne muscular dystrophy cardiomyopathy. Hum. Mol. Genet. 30 (23), 2347–2361. 10.1093/hmg/ddab199 34270708 PMC8600005

[B52] Gartz M. Darlington A. Afzal M. Z. Strande J. L. (2018). Exosomes exert cardioprotection in dystrophin-deficient cardiomyocytes via ERK1/2-p38/MAPK signaling. Sci. Rep. 8 (1), 16519. 10.1038/s41598-018-34879-6 30410044 PMC6224575

[B53] Gartz M. Lin C.-W. Sussman M. A. Lawlor M. W. Strande J. L. (2020). Duchenne muscular dystrophy (DMD) cardiomyocyte-secreted exosomes promote the pathogenesis of DMD-associated cardiomyopathy. Dis. Models Mech. 13 (11), 045559. 10.1242/dmm.045559 PMC7673361 33188007

[B54] Guay C. Regazzi R. (2017). Exosomes as new players in metabolic organ cross-talk. Diabetes, Obes. Metabolism 19 (S1), 137–146. 10.1111/dom.13027 28880477

[B55] Guescini M. Canonico B. Lucertini F. Maggio S. Annibalini G. Barbieri E. (2015). Muscle releases alpha-sarcoglycan positive extracellular vesicles carrying miRNAs in the bloodstream. PLOS One 10 (5), e0125094. 10.1371/journal.pone.0125094 25955720 PMC4425492

[B56] Guescini M. Maggio S. Ceccaroli P. Battistelli M. Annibalini G. Piccoli G. (2017). Extracellular vesicles released by oxidatively injured or intact C2C12 myotubes promote distinct responses converging toward myogenesis. INernational J. Mol. Sci. 18 (11), 2488. 10.3390/ijms18112488 PMC5713454 29165341

[B57] Guiraud S. Davies K. E. (2017). Pharmacological advances for treatment in Duchenne muscular dystrophy. Curr. Opin. Pharmacol. 34, 36–48. 10.1016/j.coph.2017.04.002 28486179

[B58] Haidet A. M. Rizo L. Handy C. Umapathi P. Eagle A. Shilling C. (2008). Long-term enhancement of skeletal muscle mass and strength by single gene administration of myostatin inhibitors. Proc. Natl. Acad. Sci. U. S. A. 105 (11), 4318–4322. 10.1073/pnas.0709144105 18334646 PMC2393740

[B59] Halkein J. Tabruyn S. P. Ricke-Hoch M. Haghikia A. Nguyen N.-Q.-N. Scherr M. (2013). MicroRNA-146a is a therapeutic target and biomarker for peripartum cardiomyopathy. J. Clin. Investigation 123 (5), 2143–2154. 10.1172/JCI64365 PMC3638905 23619365

[B60] Heier C. R. Damsker J. M. Yu Q. Dillingham B. C. Huynh T. Van der Meulen J. H. (2013). VBP15, a novel anti-inflammatory and membrane-stabilizer, improves muscular dystrophy without side effects. EMBO Mol. Med. 5, 1569–1585. 10.1002/emmm.201302621 24014378 PMC3799580

[B61] Heijnen H. F. Schiel A. E. Fijnheer R. Geuze H. J. Sixma J. J. (1999). Activated platelets release two types of membrane vesicles: Microvesicles by surface shedding and exosomes derived from exocytosis of multivesicular bodies and alpha-granules. Blood 94 (11), 3791–3799. 10.1182/blood.v94.11.3791.423a22_3791_3799 10572093

[B62] Hoffman E. P. Brown R. H. Jr Kunkel L. M. (1987a). Dystrophin: The protein product of the Duchenne muscular dystrophy locus. Cell 51 (6), 919–928. 10.1016/0092-8674(87)90579-4 3319190

[B63] Hoffman E. P. Monaco A. P. Feener C. C. Kunkel L. M. (1987b). Conservation of the Duchenne muscular dystrophy gene in mice and humans. Science 238 (4825), 347–350. 10.1126/science.3659917 3659917

[B64] Huotari J. Helenius A. (2011). Endosome maturation. EMBO J. 30, 3481–3500. 10.1038/emboj.2011.286 21878991 PMC3181477

[B65] Ibrahim A. G.-E. Cheng K. Marbán E. (2014). Exosomes as critical agents of cardiac regeneration triggered by cell therapy. Stem Cell Rep. 2 (5), 606–619. 10.1016/j.stemcr.2014.04.006 PMC4050492 24936449

[B66] Infanger D. W. Cao X. Butler S. D. Burmeister M. A. Zhou Y. Stupinski J. A. (2010). Silencing nox4 in the paraventricular nucleus improves myocardial infarction-induced cardiac dysfunction by attenuating sympathoexcitation and periinfarct apoptosis. Circulation Res. 106 (11), 1763–1774. 10.1161/CIRCRESAHA.109.213025 20413786 PMC2887701

[B67] Jalabert A. Vial G. Guay C. Wiklander O. P. Nordin J. Z. Aswad H. (2016). Exosome-like vesicles released from lipid-induced insulin-resistant muscles modulate gene expression and proliferation of beta recipient cells in mice. Diabetologia 59 (5), 1049–1058. 10.1007/s00125-016-3882-y 26852333

[B68] Jansson M. D. Damas N. D. Lees M. Jacobsen A. Lund A. H. (2015). miR-339-5p regulates the p53 tumor-suppressor pathway by targeting MDM2. Oncogene 34, 1908–1918. 10.1038/onc.2014.130 24882579

[B69] Jeanson-Leh L. Lameth J. Krimi S. Buisset J. Amor F. Le Guiner C. (2014). Serum profiling identifies novel muscle miRNA and cardiomyopathy-related miRNA biomarkers in golden retriever muscular dystrophy dogs and Duchenne muscular dystrophy patients. Pthology 184 (11), 2885–2898. 10.1016/j.ajpath.2014.07.021 25194663

[B70] Keefe A. C. Kardon G. (2015). A new role for dystrophin in muscle stem cells. Nat. Med. 21, 1391–1393. 10.1038/nm.4006 26646493

[B71] Kervadec A. Bellamy V. El Harane N. Arakélian L. Vanneaux V. Cacciapuoti I. (2016). Cardiovascular progenitor-derived extracellular vesicles recapitulate the beneficial effects of their parent cells in the treatment of chronic heart failure. J. Heart Lung Translplantation Official Publ. Int. Soc. Heart Transplant. 35 (6), 795–807. 10.1016/j.healun.2016.01.013 27041495

[B72] Klingler W. Jurkat-Rott K. Lehmann-Horn F. Schleip R. (2012). The role of fibrosis in Duchenne muscular dystrophy. Acta Myol. 31 (3), 184–195. 23620650 PMC3631802

[B73] Korkut C. Ataman B. Ramachandran P. Ashley J. Barria R. Gherbesi N. (2009). Trans-synaptic transmission of vesicular Wnt signals through Evi/Wntless. Cell 139 (2), 393–404. 10.1016/j.cell.2009.07.051 19837038 PMC2785045

[B74] Kunkel L. Hejtmancik J. Caskey C. Speer A. Monaco A. Middlesworth W. (1986). Analysis of deletions in DNA from patients with Becker and Duchenne muscular dystrophy. Nature 322 (6074), 73–77. 10.1038/322073a0 3014348

[B75] Lai C. R. Arslan F. Lee M. M. Sze N. S. Choo A. Chen T. S. (2010). Exosome secreted by MSC reduces myocardial ischemia/reperfusion injury. Stem Cell Res. 4 (3), 214–222. 10.1016/j.scr.2009.12.003 20138817

[B76] Landfeldt E. Thompson R. Sejersen T. McMillan H. J. Kirschner J. Lochmüller H. (2020). Life expectancy at birth in Duchenne muscular dystrophy: A systematic review and meta-analysis. Eur. J. Epidemiol. 35 (7), 643–653. 10.1007/s10654-020-00613-8 32107739 PMC7387367

[B77] Leal L. G. Lopes M. A. Batista M. L. Jr. (2018). Physical exercise-induced myokines and muscle-adipose tissue crosstalk: A review of current knowledge and the implications for health and metabolic diseases. Front. Physiology 9, 1307. 10.3389/fphys.2018.01307 PMC6166321 30319436

[B78] Lee A. Moon J. Kho C. (2022). MicroRNAs in dystrophinopathy. Int. J. Mol. Sci. 23, 7785. 10.3390/ijms23147785 35887128 PMC9318410

[B79] Lee S. Vasudevan S. (2013). Post-transcriptional stimulation of gene expression by microRNAs. Adv. Exp. Med. Biol. 768, 97–126. 10.1007/978-1-4614-5107-5_7 23224967

[B80] Leng L. Dong X. Gao X. Ran N. Geng M. Zuo B. (2021). Exosome-mediated improvement in membrane integrity and muscle function in dystrophic mice. Mol. Ther. J. Am. Soc. Gene Ther. 29 (4), 1459–1470. 10.1016/j.ymthe.2020.12.018 PMC8058444 33333294

[B81] Levi O. Genin O. Angelini C. Halevy O. Pines M. (2015). Inhibition of muscle fibrosis results in increases in both utrophin levels and the number of revertant myofibers in Duchenne muscular dystrophy. Oncotarget 6 (27), 23249–23260. 10.18632/oncotarget.4021 26015394 PMC4695115

[B82] Li J. Salvador A. M. Li G. Valkov N. Ziegler O. Yeri A. (2021). Mir-30d regulates cardiac remodeling by intracellular and paracrine signaling. Circulation Res. 128 (1), e1–e23. 10.1161/CIRCRESAHA.120.317244 33092465 PMC7790887

[B83] Lin B. Li Y. Han L. Kaplan A. D. Ao Y. Kalra S. (2015). Modeling and study of the mechanism of dilated cardiomyopathy using induced pluripotent stem cells derived from individuals with Duchenne muscular dystrophy. Dis. Models Mech. 8 (5), 457–466. 10.1242/dmm.019505 PMC4415895 25791035

[B84] Lipkin D. P. Jones D. A. Round J. M. Poole-Wilson P. A. (1988). Abnormalities of skeletal muscle in patients with chronic heart failure. Int. J. Cardiol. 18 (2), 187–195. 10.1016/0167-5273(88)90164-7 2830194

[B85] Liu Z. Lu C.-L. Cui L.-P. Hu Y.-L. Yu Q. Jiang Y. (2012). MicroRNA-146a modulates TGF-β1-induced phenotypic differentiation in human dermal fibroblasts by targeting SMAD4. Archives Dermatological Res. 304 (3), 195–202. 10.1007/s00403-011-1178-0 21968601

[B86] Logozzi M. Mizzoni D. Di Raimo R. Hiuliani A. Maggi M. Sciarra A. (2021). Plasmatic exosome number and size distinguish prostate cancer patients from healthy individuals: A prospective clinical study. Front. Oncol. 11, 727317. 10.3389/fonc.2021.727317 34745949 PMC8564386

[B87] Ma H. Ye P. Zhang A.-k. Yu W.-d. Lin S. Zheng Y.-g. (2022). Upregulation of miR-335-5p contributes to right ventricular remodeling via calumenin in pulmonary arterial hypertension. BioMed Res. Int. 2022, 9294148. 10.1155/2022/9294148 36246958 PMC9557250

[B88] Malik Z. A. Kott K. S. Poe A. J. Kuo T. Chen L. Ferrara K. W. (2013). Cardiac myocyte exosomes: Stability, HSP60, and proteomics. Am. J. Physiology Heart Circulatory Physiology 304 (7), H954–H965. 10.1152/ajpheart.00835.2012 PMC3625894 23376832

[B89] Marbán E. (2018). A mechanistic roadmap for the clinical application of cardiac cell therapies. Nat. Biomed. Eng. 2, 353–361. 10.1038/s41551-018-0216-z 30740264 PMC6366940

[B90] Marini V. Marino F. Aliberti F. Giarratana N. Pozzo E. Duelen R. (2022). Long-term culture of patient-derived cardiac organoids recapitulated Duchenne muscular dystrophy cardiomyopathy and disease progression. Front. Cell Dev. Biol. 10, 878311. 10.3389/fcell.2022.878311 36035984 PMC9403515

[B91] Mathivanan S. Ji H. Simpson R. J. (2010). Exosomes: Extracellular organelles important in intercellular communication. J. Proteomics 73 (10), 1907–1920. 10.1016/j.jprot.2010.06.006 20601276

[B92] Matsuzaka Y. Hirai Y. Hashido K. Okada T. (2022). Therapeutic application of extracellular vesicles-capsulated adeno-associated virus vector via nSMase2/smpd3, satellite, and immune cells in Duchenne muscular dystrophy. Int. J. Mol. Sci. 23 (3), 1551. 10.3390/ijms23031551 35163475 PMC8836108

[B93] Matsuzaka Y. Tanihata J. Komaki H. Ishiyama A. Oya Y. Rüegg U. (2016). Characterization and functional analysis of extracellular vesicles and muscle-abundant miRNAs (miR-1, miR-133a, and miR-206) in C2C12 myocytes and mdx mice. PLOS One 11 (12), e0167811. 10.1371/journal.pone.0167811 27977725 PMC5158003

[B94] Matsuzaka Y. Tanihata J. Ooshima Y. Yamada D. Sekiguchi M. Miyatake S. (2020). The nSMase2/Smpd3 gene modulates the severity of muscular dystrophy and the emotional stress response in mdx mice. BMC Med. 18 (1), 343. 10.1186/s12916-020-01805-5 33208172 PMC7677854

[B95] McDonald C. (2018). A phase 2, randomized, double-blind, placebo-controlled trial evaluating the safety and efficacy of intravenous delivery of allogeneic cardiosphere-derived cells in subjects with Duchenne muscular dystrophy. Available at: https://clinicaltrials.gov/ct2/show/NCT03406780?term=cardiosphere&cond=Muscular+Dystrophies&draw=2&rank=3 .

[B96] McDonald C. (2022). A phase 3, randomized, double-blind, placebo-controlled trial evaluating the efficacy and safety of human allogeneic cardiosphere-derived cells for the treatment of Duchenne muscular dystrophy. Available at: https://clinicaltrials.gov/ct2/show/NCT05126758?term=exosome&cond=Muscular+Dystrophies&draw=2&rank=1 .

[B97] McDonald C. M. Marbán E. Hendrix S. Hogan N. Smith R. R. Eagle M. (2022). Repeated intravenous cardiosphere-derived cell therapy in late-stage Duchenne muscular dystrophy (HOPE-2): A multicentre, randomised, double-blind, placebo-controlled, phase 2 trial. Lancet 399 (10329), 1049–1058. 10.1016/S0140-6736(22)00012-5 35279258

[B98] Meldolesi J. (2021). Extracellular vesicles (exosomes and ectosomes) play key roles in the pathology of brain diseases. Mol. Biomed. 2, 18. 10.1186/s43556-021-00040-5 35006460 PMC8607397

[B99] Menasché P. (2022). Human embryonic stem cells still have a place in the cell therapy landscape. Cardiovasc. Res. 118, e96–e97. 10.1093/cvr/cvac117 35859007

[B100] Menasché P. Vanneaux V. Hagège A. Bel A. Cholley B. Parouchev A. (2018). Transplantation of human embryonic stem cell-derived cardiovascular progenitors for severe ischemic left ventricular dysfunction. J. Am. Coll. Cardiol. 71 (4), 429–438. 10.1016/j.jacc.2017.11.047 29389360

[B101] Meng Q. Zhang J. Zhong J. Zeng D. Lan D. (2022). Novel miRNA biomarkers for patients with Duchenne muscular dystrophy. Front. Neurology 13, 921785. 10.3389/fneur.2022.921785 PMC9298557 35873767

[B102] Messina E. De Angelis L. Frati G. Morrone S. Chimenti S. Fiordaliso F. (2004). Isolation and expansion of adult cardiac stem cells from human and murine heart. Circulation Res. 95 (9), 911–921. 10.1161/01.RES.0000147315.71699.51 15472116

[B103] Meyers T. A. Townsend D. (2019). Cardiac pathophysiology and the future of cardiac therapies in Duchenne muscular dystrophy. Int. J. Mol. Sci. 20 (17), 4098. 10.3390/ijms20174098 31443395 PMC6747383

[B104] Monaco A. P. Neve R. L. Colletti-Feener C. Bertelson C. J. Kurnit D. M. Kunkel L. M. (1986). Isolation of candidate cDNAs for portions of the Duchenne muscular dystrophy gene. Nature 323 (6089), 646–650. 10.1038/323646a0 3773991

[B105] Murach K. A. McCarthy J. J. (2017). MicroRNAs, heart failure, and aging: Potential interactions with skeletal muscle. Heart Fail. Rev. 22 (2), 209–218. 10.1007/s10741-016-9572-5 27384434 PMC5582593

[B106] Nag A. C. (1980). Study of non-muscle cells of the adult mammalian heart: A fine structural analysis and distribution. Cytobios 28 (109), 41–61. 7428441

[B107] Nguyen B. Y. Azam T. Wang X. (2021). Cellular signaling cross-talk between different cardiac cell populations: An insight into the role of exosomes in the heart diseases and therapy. Am. J. Physiology Heart Circulatory Physiology 320 (4), H1213–H1234. 10.1152/ajpheart.00718.2020 PMC8260388 33513083

[B108] Okubo M. Noguchi S. Hayashi S. Nakamura H. Komaki H. Matsuo M. (2020). Exon skipping induced by nonsense/frameshift mutations in DMD gene results in Becker muscular dystrophy. Hum. Genet. 139 (2), 247–255. 10.1007/s00439-019-02107-4 31919629 PMC6981323

[B109] Perbellini F. Watson S. A. Bardi I. Terracciano C. M. (2018). Heterocellularity and cellular cross-talk in the cardiovascular system. Front. Cardiovasc. Med. 5, 143. 10.3389/fcvm.2018.00143 30443550 PMC6221907

[B110] Petrillo S. Pelosi L. Piemonte F. Travaglini L. Forcina L. Catteruccia M. (2017). Oxidative stress in Duchenne muscular dystrophy: Focus on the NRF2 redox pathway. Hum. Mol. Genet. 26 (14), 2781–2790. 10.1093/hmg/ddx173 28472288

[B111] Pironti G. Strachan R. T. Abraham D. Yu S. M.-W. Chen M. Chen W. (2015). Circulating exosomes induced by cardiac pressure overload contain functional angiotensin II type 1 receptors. Circulation 131 (24), 2120–2130. 10.1161/CIRCULATIONAHA.115.015687 25995315 PMC4470842

[B112] Ribeiro A. F. Jr Souza L. S. Almeida C. F. Ishiba R. Fernandes S. A. Guerrueri D. A. (2019). Muscle satellite cells and impaired late stage regeneration in different murine models for muscular dystrophies. Sci. Rep. 9 (1), 11842. 10.1038/s41598-019-48156-7 31413358 PMC6694188

[B113] Rogers R. G. Fournier M. Sanchez L. Ibrahim A. G. Aminzadeh M. A. Lewis M. I. (2019). Disease-modifying bioactivity of intravenous cardiosphere-derived cells and exosomes in mdx mice. JCI Insight 4 (7), e125754. 10.1172/jci.insight.125754 30944252 PMC6483717

[B114] Rome S. Forterre A. Mizgier M. L. Bouzakri K. (2019). Skeletal muscle-released extracellular vesicles: State of the art. Front. Physiology 10, 929. 10.3389/fphys.2019.00929 PMC6695556 31447684

[B115] Rome S. (2022). Muscle and adipose tissue communicate with extracellular vesicles. Int. J. Mol. Sci. 23, 7052. 10.3390/ijms23137052 35806052 PMC9266961

[B116] Rotini A. Martínez-Sarrà E. Pozzo E. Sampaolesi M. (2018). Interactions between microRNAs and long non-coding RNAs in cardiac development and repair. Pharmacol. Res. 127, 58–66. 10.1016/j.phrs.2017.05.029 28629929

[B117] Saheera S. Jani V. P. Witwer K. W. Kutty S. (2021). Extracellular vesicle interplay in cardiovascular pathophysiology. Am. J. Physiology Heart Circulatory Physiology 320 (5), H1749–H1761. 10.1152/ajpheart.00925.2020 PMC8163654 33666501

[B118] Salam E. A. Abdel-Meguid I. Korraa S. (2007). Markers of oxidative stress and aging in Duchene muscular dystrophy patients and the possible ameliorating effect of He:Ne laser. Acta Myol. 26 (1), 14–21. 17915565 PMC2949317

[B119] Samani A. Hightower R. M. Reid A. L. English K. G. Lopez M. A. Doyle J. S. (2022). miR-486 is essential for muscle function and suppresses a dystrophic transcriptome. Life Sci. Alliance 5 (9), e202101215. 10.26508/lsa.202101215 35512829 PMC9087951

[B120] Sampaolesi M. Blot S. D'Antona G. Granger N. Tonlorenzi R. Innocenzi A. (2006). Mesoangioblast stem cells ameliorate muscle function in dystrophic dogs. Nature 444, 574–579. 10.1038/nature05282 17108972

[B121] Shirokova N. Niggli E. (2013). Cardiac phenotype of Duchenne muscular dystrophy: Insights from cellular studies. J. Mol. Cell. Cardiol. 58, 217–224. 10.1016/j.yjmcc.2012.12.009 23261966 PMC3615054

[B122] Silva I. S. Pedrosa R. Azevedo I. G. Forbes A.-M. Fregonezi G. A. Dourado Junior M. E. (2019). Respiratory muscle training in children and adults with neuromuscular disease. Cochrane Database Syst. Rev. 9 (9), CD011711. 10.1002/14651858.CD011711.pub2 31487757 PMC6953358

[B123] Skog J. Würdinger T. van Rijn S. Meijer D. H. Gainche L. Sena-Esteves M. (2008). Glioblastoma microvesicles transport RNA and proteins that promote tumour growth and provide diagnostic biomarkers. Nat. Cell Biol. 10 (12), 1470–1476. 10.1038/ncb1800 19011622 PMC3423894

[B124] Smith R. R. Barile L. Cho H. C. Leppo M. K. Hare J. M. Messina E. (2007). Regenerative potential of cardiosphere-derived cells expanded from percutaneous endomyocardial biopsy specimens. Circulation 115 (7), 896–908. 10.1161/CIRCULATIONAHA.106.655209 17283259

[B125] Subra C. Laulagnier K. Perret B. Record M. (2007). Exosome lipidomics unravels lipid sorting at the level of multivesicular bodies. Biochimie 89 (2), 205–212. 10.1016/j.biochi.2006.10.014 17157973

[B126] Sullivan M. J. Green H. J. Cobb F. R. (1990). Skeletal muscle biochemistry and histology in ambulatory patients with long-term heart failure. Circulation 81 (2), 518–527. 10.1161/01.cir.81.2.518 2297859

[B127] Tang L. Xie J. Yu X. Zheng Y. (2020). MiR-26a-5p inhibits GSK3β expression and promotes cardiac hypertrophy *in vitro* . PeerJ 8, e10371. 10.7717/peerj.10371 33240671 PMC7678492

[B128] Tao L. Bei Y. Zhou Y. Xiao J. Li X. (2015). Non-coding RNAs in cardiac regeneration. Oncotarget 6, 42613–42622. 10.18632/oncotarget.6073 26462179 PMC4767457

[B129] Trajkovic K. Hsu C. Chiantia S. Rajendran L. Wenzel D. Wieland F. (2008). Ceramide triggers budding of exosome vesicles into multivesicular endosomes. Science 319 (5867), 1244–1247. 10.1126/science.1153124 18309083

[B130] Tseliou E. Fouad J. Reich H. Slipczuk L. de Couto G. Aminzadeh M. (2015). Fibroblasts rendered antifibrotic, antiapoptotic, and angiogenic by priming with cardiosphere-derived extracellular membrane vesicles. J. Am. Coll. Cardiol. 66 (6), 599–611. 10.1016/j.jacc.2015.05.068 26248985 PMC4593504

[B131] Valadares M. Gomes J. Castello G. Assoni A. Pellati M. Bueno C. (2014). Human adipose tissue derived pericytes increase life span in utrn tm1Ked dmd mdx/J mice. Stem Cell Rev. Rep. 10, 830–840. 10.1007/s12015-014-9537-9 24943487

[B132] Valadi H. Ekström K. Bossios A. Sjöstrand M. Lee J. J. Lötvall J. O. (2007). Exosome-mediated transfer of mRNAs and microRNAs is a novel mechanism of genetic exchange between cells. Nat. Cell Biol. 9 (6), 654–659. 10.1038/ncb1596 17486113

[B133] Vasa-Nicotera M. Chen H. Tucci P. Yang A. L. Saintigny G. Menghini R. (2011). miR-146a is modulated in human endothelial cell with aging. Atherosclerosis 217 (2), 326–330. 10.1016/j.atherosclerosis.2011.03.034 21511256

[B134] Vechetti I. J. Jr Valentino T. Mobley C. B. McCarthy J. J. (2020). The role of extracellular vesicles in skeletal muscle and systematic adaptation to exercise. J. Physiology 599 (3), 845–861. 10.1113/JP278929 PMC7363579 31944292

[B135] Verhaart I. E. Aartsma-Rus A. (2019). Therapeutic developments for Duchenne muscular dystrophy. Nat. Rev. Neurol. 15, 373–386. 10.1038/s41582-019-0203-3 31147635

[B136] Villa F. Quarto R. Tasso R. (2019). Extracellular vesicles as natural, safe and efficient drug delivery systems. Pharmaceutics 11 (11), 557. 10.3390/pharmaceutics11110557 31661862 PMC6920944

[B137] Wagner K. T. Radisic M. (2021). A new role for extracellular vesicles in cardiac tissue engineering and regenerative medicine. Adv. NanoBiomed Res. 1 (11), 2100047. 10.1002/anbr.202100047 34927167 PMC8680295

[B138] Wakayama Y. Schotland D. L. Bonilla E. Orecchio E. (1979). Quantitative ultrastructural study of muscle satellite cells in Duchenne dystrophy. Neurology 29 (3), 401–407. 10.1212/wnl.29.3.401 571989

[B139] Waldenström A. Gennebäck N. Hellman U. Ronquist G. (2012). Cardiomyocyte microvesicles contain DNA/RNA and convey biological messages to target cells. PLOS One 7 (4), e34653. 10.1371/journal.pone.0034653 22506041 PMC3323564

[B140] Wang L. Zhou L. Jiang P. Lu L. Chen X. Lan H. (2012). Loss of miR-29 in myoblasts contributes to dystrophic muscle pathogenesis. Mol. Ther. J. Am. Soc. Gene Ther. 20 (6), 1222–1233. 10.1038/mt.2012.35 PMC3369280 22434133

[B141] Wang W. Li M. Chen Z. Xu L. Chang M. Wang K. (2022). Biogenesis and function of extracellular vesicles in pathophysiological processes of skeletal muscle atrophy. Biochem. Pharmacol. 198, 114954. 10.1016/j.bcp.2022.114954 35167807

[B142] Wang X. Ha T. Liu L. Zou J. Zhang X. Kalbfleisch J. (2013). Increased expression of microRNA-146a decreases myocardial ischaemia/reperfusion injury. Cardiovasc. Res. 97 (3), 432–442. 10.1093/cvr/cvs356 23208587 PMC3567787

[B143] Wang X. Huang W. Liu G. Cai W. Millard R. W. Wang Y. (2014). Cardiomyocytes mediate anti-angiogenesis in type 2 diabetic rats through the exosomal transfer of miR-320 into endothelial cells. J. Mol. Cell. Cardiol. 74, 139–150. 10.1016/j.yjmcc.2014.05.001 24825548 PMC4120246

[B144] Yang B. Lin H. Xiao J. Lu Y. Luo X. Li B. (2007). The muscle-specific microRNA miR-1 regulates cardiac arrhythmogenic potential by targeting GJA1 and KCNJ2. Nat. Med. 13 (4), 486–491. 10.1038/nm1569 17401374

[B145] Yang Z. Zingarelli B. Szabó C. (2000). Crucial role of endogenous interleukin-10 production in myocardial ischemia/reperfusion injury. Circulation 101 (9), 1019–1026. 10.1161/01.cir.101.9.1019 10704170

[B146] Yao S. Chen Z. Yu Y. Zhang N. Jiang H. Zhang G. (2021). Current pharmacological strategies for Duchenne muscular dystrophy. Front. Cell Dev. Biol. 9, 689533. 10.3389/fcell.2021.689533 34490244 PMC8417245

[B147] Yates A. G. Pink R. C. Erdbrügger U. Siljander P. R.-M. Dellar E. R. Pantazi P. (2022). In sickness and in health: The functional role of extracellular vesicles in physiology and pathology *in vivo*: Part II: Pathology: Part II: Pathology. J. Extracell. Vesicles 11 (1), e12190. 10.1002/jev2.12190 35041301 PMC8765328

[B148] Yedigaryan L. Gatti M. Marini V. Maraldi T. Sampaolesi M. (2022a). Shared and divergent epigenetic mechanisms in cachexia and sarcopenia. Cells 11 (15), 2293. 10.3390/cells11152293 35892590 PMC9332174

[B149] Yedigaryan L. Martínez-Sarrà E. Giacomazzi G. Giarratana N. van der Veer B. K. Rotini A. (2022b). Extracellular vesicle-derived miRNAs improve stem cell-based therapeutic approaches in muscle wasting conditions. Front. Immunol. 13, 977617. 10.3389/fimmu.2022.977617 36451814 PMC9702803

[B150] Yedigaryan L. Sampaolesi M. (2021). Therapeutic implications of miRNAs for muscle-wasting conditions. Cells 10 (11), 3035. 10.3390/cells10113035 34831256 PMC8616481

[B151] Young C. S. Hicks M. R. Ermolova N. V. Nakano H. Jan M. Younesi S. (2016). A single CRISPR-cas9 deletion strategy that targets the majority of DMD patients restores dystrophin function in hiPSC-derived muscle cells. Cell Stem Cell 18 (4), 533–540. 10.1016/j.stem.2016.01.021 26877224 PMC4826286

[B152] Yu D.-W. Ge P.-P. Liu A.-L. Yu X.-Y. Liu T.-T. (2019). HSP20-mediated cardiomyocyte exosomes improve cardiac function in mice with myocardial infarction by activating Akt signaling pathway. Eur. Rev. Med. Pharmacol. Sci. 23 (11), 4873–4881. 10.26355/eurrev_201906_18075 31210321

[B153] Yu X. Deng L. Wang D. Li N. Chen X. Cheng X. (2012). Mechanism of TNF-α autocrine effects in hypoxic cardiomyocytes: Initiated by hypoxia inducible factor 1α, presented by exosomes. J. Mol. Cell. Cardiol. 53 (6), 848–857. 10.1016/j.yjmcc.2012.10.002 23085511

[B154] Zanotti S. Gibertini S. Blasevich F. Bragato C. Ruggieri A. Saredi S. (2018). Exosomes and exosomal miRNAs from muscle-derived fibroblasts promote skeletal muscle fibrosis. Matrix Biol. J. Int. Soc. Matrix Biol. 74, 77–100. 10.1016/j.matbio.2018.07.003 29981373

[B155] Zanotti S. Gibertini S. Curcio M. Savadori P. Pasanisi B. Morandi L. (2015). Opposing roles of miR-21 and miR-29 in the progression of fibrosis in Duchenne muscular dystrophy. Biochimica Biophysica Acta 1852 (7), 1451–1464. 10.1016/j.bbadis.2015.04.013 25892183

[B156] Zardi E. M. Abbate A. Zardi D. M. Dobrina A. Margiotta D. Van Tassell B. W. (2010). Cirrhotic cardiomyopathy. J. Am. Coll. Cardiol. 56 (7), 539–549. 10.1016/j.jacc.2009.12.075 20688208

[B157] Zhang C. Liu J. Wang X. Wu R. Lin M. Laddha S. V. (2014). MicroRNA-339-5p inhibits colorectal tumorigenesis through regulation of the MDM2/p53 signaling. Oncotarget 5 (19), 9106–9117. 10.18632/oncotarget.2379 25193859 PMC4253422

[B158] Zhang X. Wang X. Zhu H. Kranias E. G. Tang Y. Peng T. (2012). Hsp20 functions as a novel cardiokine in promoting angiogenesis via activation of VEGFR2. PLOS One 7 (3), e32765. 10.1371/journal.pone.0032765 22427880 PMC3299679

[B159] Zhu P. Li H. Zhang A. Li Z. Zhang Y. Ren M. (2022). MicroRNAs sequencing of plasma exosomes derived from patients with atrial fibrillation: miR-124-3p promotes cardiac fibroblast activation and proliferation by regulating AXIN1. J. Physiology Biochem. 78 (1), 85–98. 10.1007/s13105-021-00842-9 34495485

